# Supplementary material for: Pharmacometabolomics Approach to Explore Pharmacokinetic Variation and Clinical Characteristics of a Single Dose of Desvenlafaxine in Healthy Volunteers
Source: Pharmaceutics. 2024 Oct 28;16(11):1385. doi: 10.3390/pharmaceutics16111385 (PMC11597518; doi:10.3390/pharmaceutics16111385)
Supplement: Supplementary file 1 [file pharmaceutics-16-01385-s001.zip › v.3 Pharmaceutics SM.pdf]

pharmaceutics

Supplementary Materials

Pharmacometabolomics Approach to Explore Pharmacokinetic Variation and Clinical Characteristics of a Single Dose of Desvenlafaxine in Healthy Volunteers

Anne Michelli Reis Silveira [1,2,†], Salvador Sánchez-Vinces [1,†], Alex Ap. Rosini Silva [1], Karen Sánchez-Luquez [1], Pedro Henrique Dias Garcia [1], Caroline de Moura Garcia [3], Rhubia Bethania Socorro Lemos de Brito [3], Ana Lais Vieira [3], Lucas Miguel de Carvalho [1], Marcia Ap. Antonio [2] and Patrícia de Oliveira Carvalho [1,*]

[1] Health Sciences Postgraduate Program, São Francisco University–USF, Bragança Paulista 12916-900, SP, Brazil; anne.silveira@unifag.com.br (A.M.R.S.); salvador.vinces@mail.usf.edu.br (S.S.-V.); alex.rosini@mail.usf.edu.br (A.A.R.S.); ksanchezluquez@gmail.com (K.S.-L.); pedroh_g@outlook.com (P.H.D.G.); lucas.miguel@usf.edu.br (L.M.d.C.)
[2] Integrated Unit of Pharmacology and Gastroenterology (UNIFAG), São Francisco University–USF, Bragança Paulista 12916-900, SP, Brazil; marcia.antonio@unifag.com.br
[3] Althaia S.A. Indústria Farmacêutica, Atibaia 12952-820, SP, Brazil; garcia.carol100@gmail.com (C.d.M.G.); rhubia.brito@althaia.com.br (R.B.S.L.d.B.); analais.vieira@althaia.com.br (A.L.V.)
* Correspondence: patricia.carvalho@usf.edu.br; Tel.: +55-11-99670-5865
† These authors contributed equally to this work.

Supplementary Files

Table S1. Demographic, clinical and laboratory characteristics. This table contains the descriptive statistics of demographic, clinical and laboratory characteristics of the participants included in the study.

Table S2. Adverse events. Reported adverse events for some participants.

Table S3. Pharmacokinetics parameters. Pharmacokinetics parameters were calculated for each participant using the concentration across time data.

Table S4. Compound and identification data. Obtained data for each compound from instrumental analysis. Putative annotation is added too.

Table S5. Pathway enrichment results. List of enriched pathways and additional statistics and data.

Table S6. Community results at pre-dose. Results of community network analysis using relative abundance at pre-dose of differential metabolites. Each module or community is composed by a metabolite linking to either a pharmacokinetic parameter or characteristic or both.

Table S7. Community results at post-dose. Results of community network analysis using relative abundance at pre-dose of differential metabolites. Each module or community are composed by a metabolite linking to either a pharmacokinetic parameter or characteristic or both.

1. Supplementary Methods

Volunteers and Study Design

The study started with 48 enrolled participants. Of these, 13 participants desisted after drug administration. Of the 35 remaining participants, 23 different adverse events were reported for some of them.

The reference formulation was desvenlafaxine succinate monohydrate controlled-release coated tablet of 100 mg Pristiq® with administration of one single-dose tablet. The volunteers were hospitalized at 7:30 p.m. and had supper before 9:30 p.m. After an overnight fast, they received (at ~5:40 a.m.) a tablet of 100 mg desvenlafaxine succinate monohydrate with 200 mL of water. The volunteers then fasted for 4 h, after which period a standard lunch was served. A specific high-calorie diet was administered thirty minutes before taking the medication; Lunch: 4 hours after taking the medication; Afternoon snack: 8 hours after taking the medication; Dinner: 10 hours after taking the medication.

It was necessary to observe the ethical principle of non-maleficence due to the adverse event of nausea being considered very common (≥ 10%). the antiemetic (Dramin® B6 DL - IV - 30 mg / 100 mL saline / 30 minutes) was administered twice: the first dose immediately after administration of the study medication and a second dose 5:00 hours after administration of the study medication.

At 2 h before and 6, 8 and 12 h after the dose administration, vital signs were checked. The recruitment period was 24 h, during which period, blood samples (8 mL) from a suitable antecubital vein were collected, post-dosing via an indwelling catheter, into Heparin lithium-containing tubes at 0, 1:00, 2:00, 3:00, 4:00, 5:00, 6:00, 6:15, 6:30, 6:45, 7:00, 7:15, 7:30, 7:45, 8:00, 8:20, 8:40, 9:00, 10:00, 12:00, 24:00, and 36:00, 48:00, 72.0h. The blood samples were centrifuged at ~2,000×g for 10 min at 4°C, and the plasma was stored at -70°C until assayed. Samples were handled under yellow light, due to the photosensitivity of the drug.

Determination of Pharmacokinetic Profile by LC-MS/MS

Chromatographic separations were performed in an LC-20AD analytical pump and SIL-20A HT autosampler from Shimadzu. The stationary phase employed a Luna AgilLuna Agilent Polaris 5 µm C18-A 50 x 2,0mm column (Phenomenex). The mobile phase consisted of a solution of 10 mM ammonium acetate with 0.1% formic acid (Phase A) and acetonitrile with 0.1% formic acid (Phase B), flowing at 0.3 mL/min in proportion (2:8). The autosampler was maintained at 22°C, with an injection volume of 10 µl and 2.2-minute duration.

Mass spectrometry was conducted on a Quattro Micro mass spectrometer from Micromass, equipped with an electrospray ionization (ESI) source. Operating in positive ionization mode with nitrogen as the desolvation gas, ESI source parameters were set as follows: source temperature of 105°C, desolvation temperature of 450°C, desolvation flow of 500 L/h, and capillary voltage of 2,00 kV. Desvalexine was detected by a multiple reaction monitoring (MRM) transition of 264.33 > 58.15 using a cone voltage of 30 V, and orphenadrine was detected by a MRM transition of 270.32 > 181.26 using a cone voltage of 20 V. Data were acquired using MassLynx 4.1.

Preparation of Standards and Quality Controls

Desvenlafaxine stock solution (108.5 µg/mL) was prepared in methanol, and diluted with H2O to obtain the corrected stock solution (100 µg/mL), for the spiking calibration curve and quality controls (QC) samples. QC samples were prepared in the following levels: low, 3 ng/mL (LQC); medium, 275 ng/mL (MQC), high, 412,5 ng/mL (HQC), and (dilution 233,75 ng/mL (DQC) (upper limit of quantification (ULOQ) added of 70% (df=4)).

Calibration curves in plasma were prepared to the final concentration range of 1, 5, 10, 50, 100, 200, 350, and 550 ng/mL. The internal standard was prepared in methanol.

Sample Extraction

Samples plasma was aliquot (300 µL) and add 25 uL of IS solution (orphenadrine) were added to the plasma, next 25 µl of HCl 1 M as well as 1,000 µl of a diethyl ether/dichloromethane (70:30 v/v) were added. Then, samples were shaken for 5 min and centrifuged at 12,000 rpm for 10 min at 4°C. Finally, the supernatant was collected and dried over a nitrogen gas (N2) flow. An acetonitrile/water solution (80:20 v/v) was used for resuspension.

Method Validation

The analytical method was validated according to Agência Nacional de Vigilância Sanitária (ANVISA, RDC N. 27/2012), observing the following parameters: specificity, carryover, matrix effect, calibration curve, accuracy, precision, and stability.

Specificity was ascertained by analyzing blank human plasma samples from six individuals and comparing the chromatograms among blank human plasma spiked with desvenlafaxine in the lower limit of quantification (LLOQ) concentration of 1ng/mL and IS. The level of hemolysis used in the validation of this study was the highest level (very low, 1; low, 2; moderate, 3; high, 4). Carryover was ascertained by analyzing three injections of the same blank sample, one sample in the LLOQ concentration (1 ng/mL), and one sample ULOQ (550 ng/mL) chromatograms. In this sequence: LLOQ sample, blank Sample, ULOQ Samples, and two blank samples.Matrix effect was ascertained by spiking eight different extracted blank human plasma samples (four normal, 2 two lipemic and 2 two hemolyzed) with desvenlafaxine at QC concentrations and the IS. Peak areas of extracted spiked samples were compared to those of standard solutions.

The calibration curve was prepared using eight different blank human plasma samples, spiked with concentrations at eight levels ranging from 1 to 550 ng/mL. Analyte concentrations in samples were calculated by linear regression equation ($y = ax + b$) where y corresponds to the analyte/IS peak area ratio and x corresponds to the ratio of desvenlafaxine to IS concentration. Due to the range of the calibration curve and lower value of the sum of the relative errors of the nominal values of the calibration versus its values obtained by the curve equation, the weighting factor of reciprocal concentration squared (1/x2) was applied. Intra- and inter-batch accuracy and precision were evaluated at five different levels (1, 3, 233.75, 275, and 412,5 ng/mL) of QC samples in quintuplicate in three different batches. The accuracy of the method was expressed as relative error (RE), whereas precision was obtained by calculating the within- and between-run coefficient of variation (CV). The acceptance criteria for RE and CV must not exceed 15% for QC and 20% for LLOQ. All stability assays were performed to cover the conditions anticipated for handling real samples: freshly prepared, post-processing, short-term, freeze-thaw, and long-term were evaluated at concentrations of LQC) and HQC). RE and CV were used to check possible variations.

Determination of Desvenlafaxine Pharmacokinetic

Following Food and Drug Administration (FDA) guidelines, blood samples were drawn up to a period of three to five times the terminal elimination half-life (t1/2) and the mean AUC0–t/AUC0–∞ ratio was required to be higher than 80%. The area under the concentration–time curve (AUC0–t) was calculated from time 0 to time t. The total area under the curve (AUC0–∞) was obtained up to the last measurable concentration and extrapolations were performed using the last measurable concentration and the terminal elimination rate constant (Ke). The terminal elimination rate constant, Ke, was estimated from the slope of the terminal log10 transformed exponentially and multiplied by −2.303

phase of the plasma of desvenlafaxine concentration–time curve (by means of the linear regression method) adjusted in the last three values. The terminal elimination half-life, t1/2, was then obtained as 0.693/Ke. The Cmax and the time to reach maximum plasma concentration (Tmax) values were determined by visual inspection of the plasma desvenlafaxine concentration–time profiles. Results are presented as mean ± standard deviation (SD).

Metabolomics Analysis

An ACQUITY UPLC was used, coupled to a XEVO-G2XS Quadrupole Time-of-Flight (QToF) mass spectrometer (Waters, Manchester, UK) equipped with an ESI source, operated both in positive (+) and negative (-) ionization modes. Chromatographic analysis was performed using an ACQUITY UPLC® CSH C18 column (C18, 2.1 mm x 100 mm x 1.7 µm, Waters), employing the mobile phase A composed of Water + 0.1% formic acid, and the mobile phase B, composed of Acetonitrile + 0.1% formic acid. The flow rate was 0.4 mL/min. Initially, the column was conditioned with 10% B and maintained for 2 min, increasing to 40% B over the next 0.5 min, increasing to 90% B over the next 4.5 min, and staying for 2 min at that rate. In 2.0 min, mobile phase B returned to 10% and was maintained for another 2.0 min, equilibrating the column for the next injection. The total running time was 13 min. The injection volume was 1 µL (+/-). The mass spectrometer was operated in MSE mode with an m/z range of 50–1200 Da, and an acquisition time of 0.5s per scan. MSE analysis was operated at 6 V for low collision energy and a ramp of 20-50 V for high collision energy. Leucine enkephalin (molecular weight = 555.62; 200 pg/mL in 1:1 ACN: H2O, v/v) was used as the lockmass for mass accuracy, and a 0.5 mM sodium formate solution was used for calibration. Other parameters were as follows: source temperature = 140 °C, desolvation temperature = 550 °C, desolvation gas flow = 900 L/h, capillary voltage = 3.0 kV (ESI+) / 2.5 kV (-), and cone voltage = 40 V. Raw data was processed using the Progenesis™ QI v2.4 software (Nonlinear Dynamics, Newcastle, UK), The LC-MS raw files were processed using the Progenesis™ QI software v2.4 (Nonlinear Dynamics, Newcastle, United Kingdom), which enabled the selection of possible adducts, peak alignment, deconvolution, and compound annotation based on MSE experiments. The adducts [M+H]+, [M+K]+, [M+Na]+, and [M+H-H2O]+ were considered for the positive acquisition mode, while [M-H]-, [M+Cl]-, [M-H2O-H]-, and [M+FA-H]- were considered for the negative acquisition mode. For each sample, Progenesis QI generates an intensity table of the ions, labeled according to their retention time and nominal masses, called features, as a function of their intensity (areas of the extracted ion chromatogram).

2. Supplementary Results

Method Validation

The selectivity analysis for desvenlafaxine indicated that the interference peaks in all blank samples were below 1% compared to the Lower Limit of Quantification (LLOQ) and IS. Chromatographic evidence showed no residual peaks in blank samples for either the analyte or the IS, confirming no carryover effect (Figure S1). The matrix effect for LQC and HQC samples was minimal at 4.74%, with no notable interference from matrix components across eight individual plasma samples.

Calibration curves demonstrated good linearity within the 1–550 ng/mL range, with individual regression equations and determination coefficients (r^2) as follows: (i) y= 0.00296424x + 0.000673827 (r^2 = 0.995983); (ii) y= 0.0034608x + 0.000175332 (r^2 = 0.997647); (iii) y= 0.00296909x - 0.0000105154 (r^2 = 0.990637). QC concentrations showed Relative Error (RE) values within ±15%. Both intra- and inter-batch precision were under 10%, indicating consistent measurements across identical concentrations. Precision and accuracy for all assays were below 15%, showcasing stability throughout the testing period. Additionally, LQC and HQC were tested for drug stability in biological fluid. The

conditions were: freshly prepared, post processing (auto-injector, 73:43 hours), short-term (72:21 hours), freeze-thaw (4 cycles) at -20° C and -70°C and long term stability at -20° C and -70°C for 90 days. All assays presented precision and accuracy values lower than 15%, demonstrating stability during the period tested.

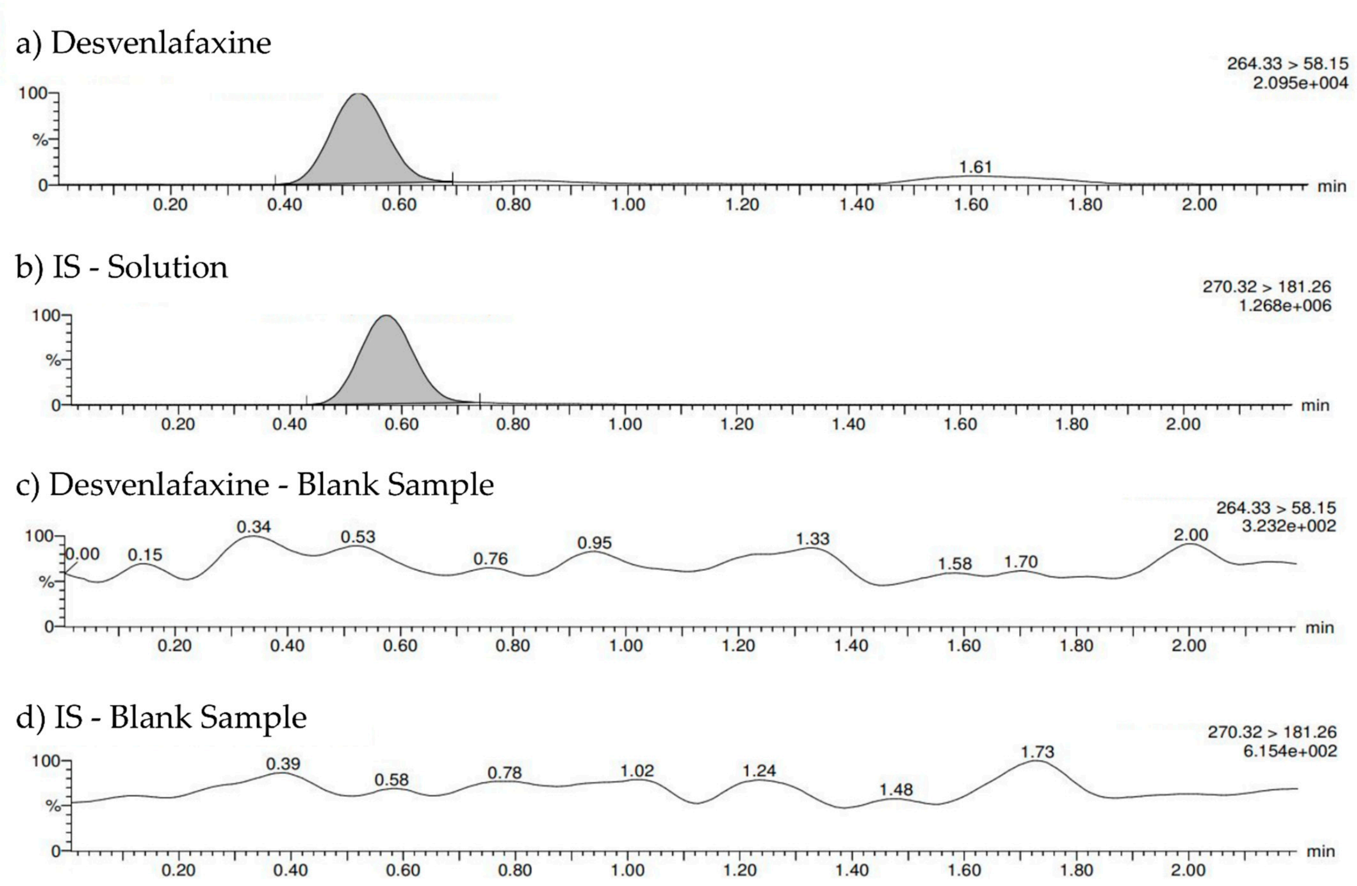

Figure S1. Chromatograms for the selectivity assay. Analysis of desvenlafaxine-spiked (a) and blank (c) plasma samples e, monitored at channel 264.3>58.2. Analysis of IS-spiked (b) and blank (d) plasma samples monitored at channel 270.3>181.2.
